# Death from Hemorrhage from Lancing the Gums

**Published:** 1853-04

**Authors:** 


### ARTICLE XVII.

### *Death from Hemorrhage from Lancing the Gums.*

Mr. Forges, in the *Lancet*, says:—Upon referring to my books, I find that, in October, 1838, I was requested to lance the gums of an infant four or five months of age, who was suffering from swelling and inflammation therein. I scarified them; and happening to pass the house about an hour afterwards, I was requested to visit the child again, as the mother informed me that the bleeding from the incision still continued. Having no styptic with me, I made use of pressure for some short time, when the bleeding seemed to be completely arrested. At about ten o'clock the same evening I was again sent for, and found that the child had suffered a rather severe loss of blood. I again resorted to pressure for some time, but without any beneficial effect; and I then cauterized the bleeding surface of the gums with the nitrate of silver; this seemed perfectly to arrest the hemorrhage. I, however, left word that I was to be sent for if anything untoward happened to my patient during the night. On riding over the following morning to see my patient, I found it on the nurse's knee, in articulo mortis, and was told that oozing from the incision had been going on most of the night; and yet they had neglected to apprise me of it, but had sent for another surgeon. What he had done I am not aware of; the child, however, died the same day.

I am afraid that such cases are not so "unique" as your correspondent seems to think; and I fear that others may have similar disasters to record.

